# Modified *in situ* Hybridization Chain Reaction Using Short Hairpin DNAs

**DOI:** 10.3389/fnmol.2020.00075

**Published:** 2020-05-12

**Authors:** Yousuke Tsuneoka, Hiromasa Funato

**Affiliations:** ^1^Department of Anatomy, Faculty of Medicine, Toho University, Tokyo, Japan; ^2^International Institutes for Integrative Sleep Medicine (WPI-IIIS), University of Tsukuba, Ibaraki, Japan

**Keywords:** short hairpin DNA, hybridization chain reaction, *in situ* hybridization, fluorophore, mouse brain, striatum, medial preoptic area

## Abstract

The visualization of multiple gene expressions in well-preserved tissues is crucial for the elucidation of physiological and pathological processes. *In situ* hybridization chain reaction (HCR) is a method to visualize specific mRNAs in diverse organisms by applying a HCR that is an isothermal enzyme-free nucleotide polymerization method using hairpin DNAs. Although *in situ* HCR is a versatile method, this method is not widely used by researchers because of their higher cost than conventional *in situ* hybridization (ISH). Here, we redesigned hairpin DNAs so that their lengths were half the length of commonly used hairpin DNAs. We also optimized the conjugated fluorophores and linkers. Modified *in situ* HCR showed sufficient fluorescent signals to detect various mRNAs such as *Penk*, *Oxtr*, *Vglut2*, *Drd1*, *Drd2*, and *Moxd1* in mouse neural tissues with a high signal-to-noise ratio. The sensitivity of modified *in situ* HCR in detecting the *Oxtr* mRNA was better than that of fluorescent ISH using tyramide signal amplification. Notably, the modified *in situ* HCR does not require proteinase K treatment so that it enables the preservation of morphological structures and antigenicity. The modified *in situ* HCR simultaneously detected the distributions of c-Fos immunoreactivity and *Vglut2* mRNA, and detected multiple mRNAs with a high signal-noise ratio at subcellular resolution in mouse brains. These results suggest that the modified *in situ* HCR using short hairpin DNAs is cost-effective and useful for the visualization of multiple mRNAs and proteins.

## Introduction

To elucidate physiological and pathological processes in living organisms, it is crucial to visualize gene expression at good spatial resolution in a well-preserved morphological context. *In situ* hybridization (ISH) is a commonly used technique for detecting specific mRNAs in cells, tissues or whole bodies (Jensen, 2014). ISH was originally developed with the use of a radioisotope-labeled antisense nucleotide (Krumlauf et al., 1987; Marcus et al., 2001), which was subsequently replaced by a digoxigenin-labeled probe that enabled alkaline phosphatase- or peroxidase-based chromogenic reactions (Funato et al., 2000; Moorman et al., 2001). The use of two or more chromogens or fluorophores in combination enables the visualization of more than one mRNA. To increase the sensitivity of the ISH method to detect less abundant mRNAs, a method called tyramide signal amplification has been developed (Zaidi et al., 2000). Recent progress in ISH includes locked nucleic acid probes that are commonly applied for the detection of small RNAs (Urbanek et al., 2015), and rolling cycle amplification that has been reported to detect a single mRNA *in situ* (Larsson et al., 2010).

Currently, enzyme-based amplification using digoxigenin-labeled probes is the most used detection technique. To visualize multiple mRNAs, however, the procedures from probe hybridization to the chromogenic reaction generally need to be conducted twice serially, which takes a substantial amount of time and requires great labor. Also, when detecting low abundance mRNAs, artificial signals are inevitably produced due to nonspecific probe hybridization and nonspecific chromogenic enzyme reactions (Jensen, 2014).

**Figure 1:**
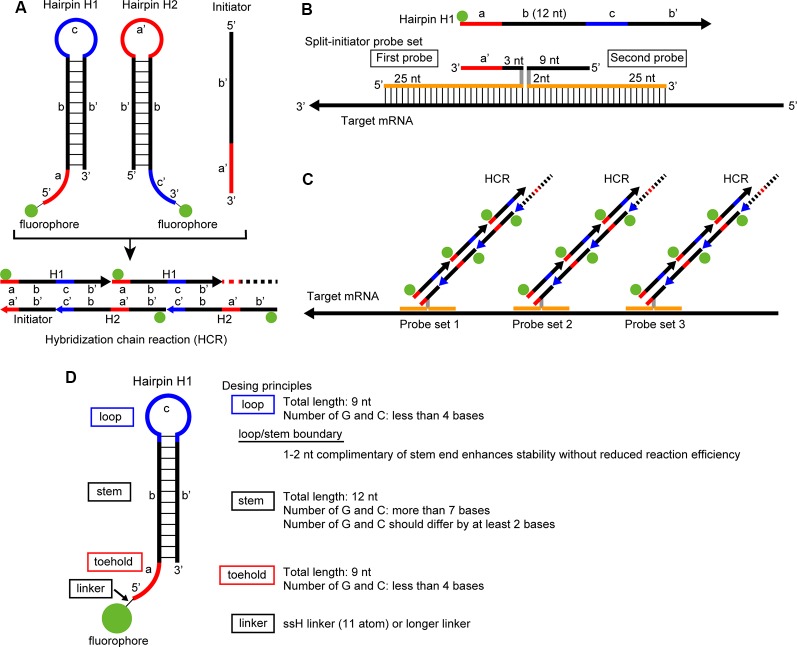
Principle of *in situ* hybridization chain reaction (HCR) and short hairpin design. **(A)** Each hairpin DNA (H1, H2) has toehold, stem and loop domains and is conjugated to a fluorophore. Whereas the sequence of the toehold domain of H1 (a) is complementary to that of the loop domain of H2 (a’), the sequence of the loop domain of H1 (c) is complementary to that of the toehold domain of H2 (c’). In the presence of an initiator that is composed of a’ and b’, the initiator hybridizes with the toehold and stem domains (a,b) of H1. Then, the remaining part of H1 (c,b’) hybridizes with the toehold and stem domains (c’,b) of H2. Thereafter, H1 and H2 continue to hybridizes with each other (lower). **(B)** Split-initiator probe set. One probe has sequence a’, first 3 nucleotide (nt) of sequence b’, 2 nt spacer sequence, and 25 nt sequence complementary to the target mRNA. Another probe has 25 nt sequence complementary to the target mRNA, 2 nt spacer sequence and the last 9 nt of sequence b’. **(C)** *In situ* HCR using split-initiator probes. Three sets of split-initiator probes hybridize with the target mRNA and lead to HCR. **(D)** Design principles of short hairpin DNA. A set of short hairpin DNAs and split-initiator probes with actual nucleotide sequences are shown in Supplementary Figure S1.

The hybridization chain reaction (HCR) is an isothermal enzyme-free polymerization method that uses two different hairpin nucleotides: H1 and H2 (Figure 1A; Dirks and Pierce, 2004). The hairpin molecules are composed of toehold, stem, and loop domains and are self-assembling and metastable in the absence of initiator nucleotides that have a specific sequence complementary to the toehold and stem domains of an H1 hairpin (Figure 1A). In the presence of an initiator nucleotide, the toehold and stem domains of an H1 hairpin hybridize with the initiator through strand displacement. The remaining single-strand part of the opened H1 hairpin that was originally the loop and stem domains, hybridizes with H2 hairpin and produces a single strand part that has a sequence identical to the initiator, which in turn hybridizes with an H1 hairpin. Therefore, once an H1 hairpin is hybridized with an initiator, the polymerization of H1 and H2 hairpins continues and forms long nicked double-helices (Figures 1A,D and Supplementary Figure S1; Dirks and Pierce, 2004).

*In situ* HCR is a method that applies the HCR technique to the visualization of a specific nucleotide in cells, tissues, and whole-mount samples using an RNA probe that has a sequence enabling the hybridization to a target mRNA and a sequence to work as an initiator of HCR (Choi et al., 2010). Choi et al. (2010) successfully visualized the localization of target mRNA in zebrafish using HCR with fluorescently labeled RNA hairpins. *In situ* HCR has been further advanced by the use of a DNA hairpin (Choi et al., 2014; Yamaguchi et al., 2015), and the use of different fluorophore-labeled DNA hairpins for the detection of multiple mRNAs (Choi et al., 2016). Although *in situ* HCR frequently has been accompanied by false-positive signals and background signals due to nonspecific probe bindings, the “third-generation” *in situ* HCR method using a pair of split probes successfully reduced nonspecific signals (Choi et al., 2018). The advantages of *in situ* HCR using split probes are a high signal-to-noise ratio, a high sensitivity that enables single-molecule imaging, and an easy protocol for multiplex staining. *In situ* HCR using split-probes does not require stringent conditions for probe hybridization, and both prehybridization and hybridization are conducted under a mild condition of 37°C, which leads to decreased damage to tissues and well-preserved morphology.

Despite the technical advantages of *in situ* HCR, this method has not become standard procedure to detect mRNAs because *in situ* HCR that uses 72-nucleotide (nt)-long DNA hairpins costs more than conventional ISH methods. The cost of oligonucleotides increases in proportion to their length, and the yield of full-length oligos decreases as their lengths increase. Thus, shorter DNA hairpins are favorable as long as they progress HCR.

In this study, we optimized and shortened DNA hairpins and initiators for *in situ* HCR with split probes. We also simplified* the in situ* HCR protocol by removing proteinase K treatment. Our modified *in situ* HCR protocol is sensitive to a low abundance mRNA, is easier to handle and enables better antigenicity preservation for immunohistochemistry at a decreased cost.

## Materials and Methods

### Design and Synthesis of DNA Probes

All nonlabeled oligo DNAs were synthesized as standard desalted oligos (Integrated DNA Technologies). We modified and optimized the design of DNA probes using short hairpin DNAs from previous studies (Choi et al., 2014, 2018) as follows. Split-initiator DNA probes were designed to minimize off-target complementarity using a homology search by NCBI Blastn^1^, and they were designed to have 45–55% GC content in their mRNA binding sites. A pair of split-initiator probes were a 39-nt long DNA (25-nt long binding sites, 2-nt long spacer, and 12-nt long split-initiator sequence) and a 36-nt long DNA (25-nt long binding sites, 2-nt-long spacer, and 9-nt-long split-initiator sequence). The split-initiator sequence of the 39-nt-long DNA has a 9-nt-long toehold and a 3-nt-long sequence that is complementary to the first 3-nt of the stem domain of the hairpins. The split-initiator sequence of the 36-nt-long DNA probe has a 9-nt-long sequence that is complementary to the following 9-nt of the stem domain of the hairpins (Figure 1B and Supplementary Figure S1). Five or 10 sets of 36-nt and 39-nt DNA probes were designed for each target mRNA (Supplementary Table S2). All probe sets for each target mRNA were prepared, mixed, and stored in TE (10 mM Tris-HCl pH 8.0 and 1 mM EDTA). Each probe mixture was subsequently purified by denaturing polyacrylamide gel electrophoresis (PAGE) using 20% polyacrylamide gels (1:40 bis and linear acrylamide). After purification, the probes were diluted in TE to 2 μM.

### Design and Synthesis of DNA Hairpin Amplifiers

To find the shortest sequences of DNA for hairpins that reliably trigger and continue HCR, we tested 36-44-nt long hairpins containing a 12-nt long stem sequence (Supplementary Table S1). The hairpin DNAs were designed using the multistate sequence design feature of NUPACK^2^, to produce target secondary structures shown in Figure 1A. After the NUPACK random design, the sequences were manually edited according to the previously reported criteria: less than 40% GC content in a toehold domain and greater than 60% GC content in a stem (Ang and Yung, 2016). The manually adjusted hairpin sequences were subsequently assessed by a NUPACK simulation to avoid undesirable secondary structures.

**Figure 2:**
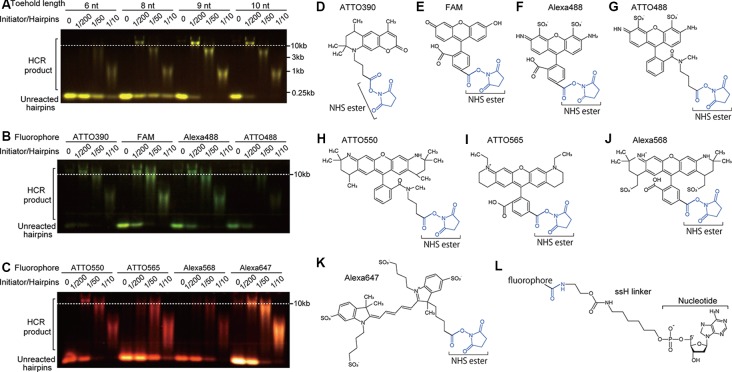
Effect of toehold length and fluorophores on HCR. **(A)** Effect of toehold length of hairpin DNAs on HCR efficiency. Short hairpin DNAs with a toehold length of 6, 8, 9, and 10 nt were examined. The ratios of initiator’s concentration to hairpin DNAs’ concentration were 0, 1/200, 1/50, and 1/10 with a constant concentration of 0.5 μM hairpin DNAs. HCR products were visualized after agarose gel electrophoresis. HCR product bands longer than 10 kb indicate efficient HCR. Unreacted hairpin DNAs were recognized as bands shorter than the position of 0.25 kb. Used hairpin DNAs were #S4 (6-nt toehold), #S8 (8-nt toehold), #S41 (9-nt toehold), and #S9 (10-nt toehold; Supplementary Table S2). **(B,C)** Effect of fluorophores on HCR efficiency. Hairpin DNAs with 9-nt toehold (#S41) conjugated with either ATTO390, FAM, Alexa488, ATTO488, ATTO550, ATTO565, ATTO568 or Alexa647 were used with different initiator/hairpin concentration ratios. Captured images taken with appropriate filter sets were overlaid based on the loading position. Dashed lines indicate a band size of 10 kb dsDNA. **(D–K)** Chemical structures of N-hydroxysuccinimide (NHS) ester (blue)-conjugated fluorophores. **(L)** Chemical structure of ssH linker that is conjugated with a fluorophore *via* an amido bond and with a nucleotide *via* a phosphoric acid.

DNA hairpins labeled at the 5′ end with an amino linker, ssH, were synthesized (FASMAC). ssH-labeling of the 5′ end of DNAs enables subsequent coupling to a fluorophore (Komatsu et al., 2008). For comparison, C6-amino linker-labeled DNAs were also prepared (FASMAC). The fluorophores that were conjugated with succinimidyl esters were FAM (Sigma–Aldrich #21878), ATTO390, ATTO488, ATTO550, ATTO565 (ATTO-TEC), Alexa Fluor488, Alexa Fluor568, and Alexa Fluor647 (Thermo Fisher Scientific; Figures 2D–K). Following chloroform purification and ethanol precipitation with MgCl2, ssH-labeled DNAs were dissolved in 0.1 M borate buffer, pH 9.0, at 1 mM concentration of the DNA. One-third volume of fluorophore-conjugated succinimidyl ester (10 mg/ml in dimethylformamide) was mixed with the DNA solution and incubated for 4 h at room temperature to allow the coupling reaction of succinimidyl ester with the ssH-amino linker conjugated to the DNA. Fluorophore-conjugated DNAs were purified by denaturing PAGE using 20% polyacrylamide gels to remove incorporated fluorophores and incorrectly synthesized shorter oligonucleotides. The fluorescence of DNA bands corresponding to expected sizes was visualized by a hand-made LED illuminator (OptoSupply) and filter sets (LEE filters) to minimize DNA damage caused by UV light; then the bands were excised from the gel. The excised gel bands were crushed and soaked in TE at 4°C overnight, and the DNA recovered by ethanol precipitation as described above. The fluorophore-labeled DNAs were eluted in TE with 150 mM NaCl, and the concentration was adjusted to 3 μM based on the 260 nm absorbance. It is noted that the exact concentration of fluorophore-labeled DNAs cannot be determined because the fluorophores absorb 260 nm light to some extent, and the stem domain (double-strand) has a lower 260 nm absorbance per nucleotide than the toehold and loop domains (single strand).

Nonlabeled DNA hairpins were used only for *in vitro* study in microtubes, not for *in situ* HCR. Nonlabeled DNAs were purified by denaturing PAGE like the process used for the labeled DNAs, except for post staining of the polyacrylamide gel using GelGreen (Biotium).

### HCR Verification of Hairpin DNAs in Microtubes

All hairpin DNAs were snap-cooled (heated to 95°C for 2 min and cooled to room temperature for 30 min) to form a hairpin structure before use. HCR was performed in 5× standard saline citrate (SSC) with 0.1% Tween 20 (5× SSCT). Each microtube was prepared by adding 2 μl of 3 μM hairpin DNAs, 6 μl of 0, 0.005, 0.02 or 0.1 μM initiator DNA, and an appropriate amount of 20× SSC with 10% Tween 20 to bring the reaction volume to 12 μl. Two hours after mixing the DNAs, the samples were supplemented with prestained loading dye (Bio-craft) and loaded into native 1% agarose gels. The gels were electrophoresed in sodium borate buffer (10 mM NaOH and 35 mM boric acid) at 135 V for 30 min and then were imaged. The intensity of each band was analyzed by ImageJ to quantify the amount of HCR products.

We determined that a pair of hairpins showed strong amplification when HCR products exceeded 10 kb with 1/200 of the initiator-to-short hairpin ratio. We also judged that initiator-independent HCR (leakage) was low when a very weak signal of the HCR product was observed after 2 h of HCR without initiator nucleotides. Also, HCR efficiency was evaluated based on the amount of unreacted hairpin (see Figure 2).

### *In situ* Hybridization

All animal procedures were conducted following the Guidelines for Animal Experiments of Toho University and were approved by the Institutional Animal Care and Use Committee of Toho University (Approved protocol ID #19-51-405). Breeding pairs of C57BL/6J mice were obtained from Japan SLC and CLEA Japan. Mice were raised in our breeding colony under controlled conditions (12 h light/dark cycle; lights on at 8:00 A.M., 23 ± 2°C, 55 ± 5% humidity; and ad libitum access to water and food). Male mice (20–30 weeks old) were used except for c-Fos staining after maternal behavior. Female mice (20–30 weeks old) were sacrificed 2 h after pup presentation for c-Fos immunohistochemistry (Tsuneoka et al., 2013). At least three mice were used for each experiment to confirm reproducibility. Mice were anesthetized with sodium pentobarbital (50 mg/kg, i.p.), and then were transcardially perfused with 4% paraformaldehyde (PFA) in phosphate-buffered saline (PBS). The brains were postfixed in 4% PFA at 4°C overnight, which was followed by cryoprotection in 30% sucrose in PBS for 2 days, embedded in Surgipath (FSC22, Leica Biosystems), and were stored at −80°C until use. The brains were cryosectioned coronally at a thickness of 40 μm.

All the sections were stained by the free-floating method, which enables better preservation of tissue morphology and uniform staining of the sections. The handling and prehybridization procedures were the same as those in our published protocol (Tsuneoka et al., 2015, 2017b). Briefly, the sections were washed with PBS containing 0.1% Tween-20 (PBST), treated with or without proteinase K (Roche, 10 mg/ml in PBST) for 10 min and postfixed with 4% PFA in PBS for 10 min at 37°C. The sections were immersed in methanol containing 0.3% H_2_O_2_ for 10 min, followed by acetylation with 0.25% acetic anhydride in 0.1 M triethanolamine (pH 8.0) for 20 min. After washing, the sections were prehybridized for 10 min at 37°C in a hybridization buffer containing 30% formamide, 10% dextran sulfate, 5× SSC, 10 mM citric acid, 0.1% Tween 20, 50 μg/ml heparin, 1× Denhardt’s solution as described (Choi et al., 2018). The sections were moved to another hybridization solution containing a mixture of 1 nM split-initiator probes, and incubated overnight at 37°C. In the case of staining for multiple targets, the probes were added simultaneously. After hybridization, the sections were washed three times for 10 min in 5× SSCT with 30% formamide at 37°C, followed by three washes for 10 min in 5× SSCT without formamide at room temperature.

For *in situ* HCR (Figure 1C), 3 μM hairpin DNA solutions were separately snap-cooled before use. The sections were incubated in amplification buffer (10% dextran sulfate in 5× SSCT) with 60 nM hairpin DNA pairs for 45 min, 2 h or overnight at 25°C. In the case of multiple staining, the hairpin DNAs were added simultaneously. Then, the samples were washed with 5× SSCT and PBST three times at room temperature.

In the case of combined ISH and immunohistochemistry, the sections were blocked using 0.8% Block Ace/PBST (Dainihon-Seiyaku), which was followed by overnight incubation with rabbit anti-c-Fos antibody (1:2,500, sc-52, Santa Cruz) in 0.4% Block Ace/PBST at 4°C. After washing three times with PBST, sections were incubated with an Alexa488-conjugated donkey anti-rabbit goat antibody (1:500, 711-545-152, Jackson ImmunoResearch) with Hoechst 33342 (1 μg/ml) for an hour at room temperature. The sections were mounted on the slide glass and cover-slipped with mounting media containing antifade (1% n-propyl gallate and 10% Mowiol4–88 in PBS).

To compare the sensitivity between *in situ* HCR and enzyme-based ISH, we also performed chromogenic ISH and fluorescent ISH using tyramide signal amplification. The cDNA fragment of *oxytocin receptor* (*Oxtr*) mRNA (GenBank ID: NM_001081147,1869-3843) was amplified, inserted into the pGEM-T plasmid (A3600, Promega), which was used into DH5α *E. coli*. After confirmation that the DNA sequence was correct, template cDNA was produced using polymerase chain reaction with specific primers (5′-ATTTAGGTGACACTATAG-3′) and (5′-TAATACGACTCACTATAGGG-3′). The probe was transcribed by SP6 RNA polymerase (P1085; Promega) in the presence of digoxigenin-labeled UTP (Dig labeling mix; Roche Diagnostics, Switzerland), which was followed by precipitation with LiCl with ethanol. The riboprobe was digested by alkaline hydrolysis to reduce the average size to 500 bases. Although hydrolyzed riboprobes sometimes increase nonspecific hybridization, our ISH protocol using a stringent wash, RNase A treatment, and non-excessive hydrolysis suppresses the background.

The prehybridization procedure was identical to that of *in situ* HCR. After acetylation, sections were washed with PBST, which was followed by incubation at 57°C overnight in a hybridization mixture containing 1 μg/ml riboprobe, 50% deionized formamide, 5× SSC (pH 7.0), 5 mM EDTA (pH 8.0), 0.2 mg/ml yeast tRNA, 0.2% Tween-20, 0.2% sodium dodecyl sulfate, 10% dextran sulfate, and 0.1 mg/ml heparin. After hybridization, the sections were washed twice with 2× SSC containing 50% formamide at 57°C for 10 min, incubated with RNAse A solution (20 μg/ml) at 37°C for 30 min, rinsed twice with 2× SSC and 0.2× SSC at 37°C (10 min each), and incubated in an alkaline phosphatase-conjugated or peroxidase-conjugated anti-digoxigenin antiserum (1:5,000 and 1:10,000, respectively; Roche) for 2 h at room temperature. Then the alkaline phosphatase-labeled sections were washed with 100 mM Tris-HCl (pH 8.0) and 150 mM NaCl and were incubated with BCIP/NBT (Roche) in 100 mM Tris-HCl (pH 9.5), 150 mM NaCl, 1 mM MgCl_2_ and 10% polyvinyl alcohol for 3 days at room temperature. The sections were dehydrated by treatment with methanol, ethanol, and xylene, and were mounted with Marinol (Muto Pure Chemical). The peroxidase-labeled sections were washed and immersed in 0.1 M boric buffer (pH 8.5) containing 10 μM Alexa568-labeled tyramide, 10% dextran sulfate, 0.05 mg/ml iodophenol and 0.003% H_2_O_2_ for 30 min. After washing, the sections were mounted as they were in the *in situ* HCR experiments.

### Histological Analysis

Fluorescent photomicrographs were obtained using a Nikon Eclipse Ni microscope equipped with the A1R confocal detection system under 20×, 40× and 100× objective lenses (Nikon Instruments Inc., Tokyo, Japan). The photomicrographs for the bright field observation were taken by a Nikon AZ-100 microscope equipped with a digital camera (Sony α7s). Images were analyzed using ImageJ software (version 1.50i, NIH, USA). Quantification of the fluorescent photographs was performed at the same threshold and adjustment of contrast.

## Results

### Reliable HCR Using 42-nt-long DNA Hairpins

To find the shortest sequences of DNA hairpins that reliably proceed with the HCR, we systematically examined hairpin DNAs with different lengths for their HCR efficiency based on the appearance of HCR products larger than 10 kb and the intensity of the unreacted hairpin DNA band. Because the commonly used DNA hairpins have toehold and loop domains of equal length and are 72-nt long containing 12-nt toehold, 24-nt stem and 12-nt loop domains (Choi et al., 2014, 2016, 2018), we first tested 7 pairs of nonlabeled 36-nt hairpin DNAs containing 6-nt toehold, 12-nt stem, and 6-nt loop domains. However, they all showed low HCR efficiency (#S1-S7 in Supplementary Table S1). Even in the presence of a high concentration of an initiator DNA, an HCR that was allowed to occur for 2 h left a majority of hairpin DNAs unreacted (Figure 2A). Thus, 36-nt hairpin DNAs were not suitable for HCR.

Next, we tested non-labeled hairpin DNAs which have 8-nt, 9-nt, or 10-nt long toehold and loop domains and 12-nt stem domains (8-nt: #S8, 9-nt: #S41, and 10-nt: #S9 in Supplementary Table S1). HCR with 8-nt long toehold and loop domains successfully produced ~10 kb products, but unreacted hairpin DNAs remained in all initiator concentrations. HCR with 9-nt and 10-nt long toehold and loop domains produced ~10 kb products, and only a small amount of hairpin DNA was unreacted (Figure 2A). There was no apparent difference in the amount of incorporated hairpin DNA between the 9-nt and 10-nt toehold/loop after a 2-h HCR, indicating that the 9-nt-long toehold and loop domains were sufficient for efficient HCR (Figure 2A, #S4, #S8–9, and #S41 in Supplementary Table S1), which is consistent with our NUPACK simulation (data not shown). Thus, we used 42-nt hairpins containing 9-nt toehold, 12-nt stem, and 9-nt loop domains thereafter.

### The Sequence of the Stem and Transition Between the Stem and Loop Affected Hairpin Stability

Since insufficient stability of hairpin DNAs leads to initiator-independent HCR, we optimized the sequence of the stem domains of short hairpin pair based on the abundance of initiator-independent HCR. Through this optimization using short hairpins with different nucleotide contents, we noticed that short hairpin pair with a similar number of G and C on one strand of the stem domain tended to result in frequent initiator-independent HCR (Table 1). In contrast, when the number difference between G and C on one strand of the stem domain is 4 or 5, the incidence of initiator-independent HCR was low (Table 1). The difference between the numbers of G and C on one strand of the stem domain significantly correlated with the initiator-independent HCR as evaluated by band intensity (Spearman’s rank correlation test, rho = −0.40, *S* = 6941.8, *p* = 0.026).

**Table 1:** Number of short hairpin pairs with different G and C number on one strand of the stem domain and their initiator-independent hybridization chain reaction (HCR).

Number difference between G and C in the stem domain*	Initiator-independent HCR				Total
	Not recognized	Faint	Mild**	Abundant***	
0	0	0	2	1	3
1	0	0	3	2	5
2	2	1	1	0	4
3	1	0	0	1	2
4	2	4	2	2	10
5	2	4	1	0	7
Total	7	9	9	6	31

Note: *For example, the stem domain sequence of GCGAGGACCACG comes to 1 because the sequence has five Gs and four Cs. **The abundance of initiator-independent HCR was less than that of unreacted hairpins. ***The abundance of initiator-independent HCR was the same or more than that of unreacted hairpins.

Also, we noticed that the shift of the transition site between the stem and loop domains by one or two nucleotides sometimes decreased initiator-independent HCR (Figure 1D). For example, the shift by one nucleotide produces a hairpin containing 9-nt toehold, 13-nt stem, and 7-nt loop domains, in which the toehold sequence hybridized with the loop domain and a part of the stem domain. However, the shift of the transition site sometimes led to decreased HCR efficiency due to the higher stability of the hairpin. Thus, the 42-nt DNA hairpins used in this study have some differences in the length of the stem and loop domains. Because of the high HCR efficiency and a small amount of initiator-independent HCR, we used #S41 hairpin DNAs to examine eight fluorophores and two linkers thereafter.

### Conjugated Fluorophores Affected HCR Efficiency

We examined which fluorophore to use affects HCR efficiency in microtubes. The conjugation of ATTO390, FAM, ATTO488, Alexa488, ATTO550, and Alexa647 (Figures 2D–K) to 42-nt DNA hairpins did not interfere with HCR, while that of ATTO565 and Alexa568 resulted in decreased HCR efficiency (Figures 2B,C). ATTO565- or Alexa568-conjugated hairpin DNAs produced a small amount of ~10 kb products with a 1:200 ratio of initiator and hairpin DNAs and left a large amount of hairpin DNAs unreacted (Figure 2C). When compared with the C6 linker, the ssH linker showed higher HCR efficiency. C6-linked ATTO550-conjugated hairpin DNA did not form ~10 kb long products with 1/200 amount of initiator DNA (Supplementary Figure S2), indicating that ssH linker is better at least for ATTO550.

### *In situ* HCR Using Short Hairpin DNAs

**Figure 3:**
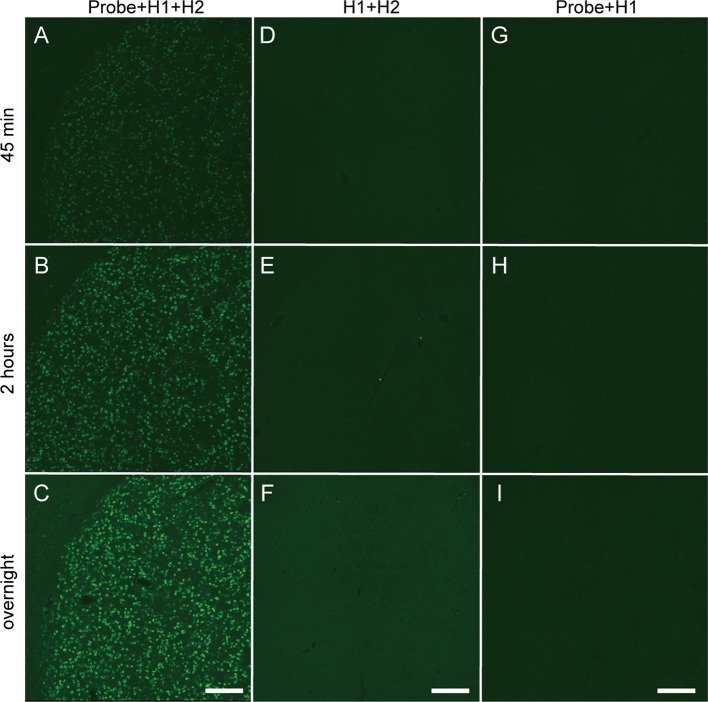
Time-dependent increase in fluorescence of *in situ* HCR using short hairpin DNAs. *Penk* mRNA expression in the mouse striatum was detected by Alexa647-conjugated hairpin DNA (#S41) and corresponding probe sets. Background signals were not subtracted. **(A–C)** Sections were hybridized with split-initiator probes followed by HCR amplification. **(D–F)** Sections were treated without probes, which was followed by HCR amplification. **(G–I)** Sections were hybridized with probes followed by only H1 hairpin DNA hybridization. **(A,D,G)** Incubation for 45 min with hairpin DNAs. **(B,E,H)** Incubation for 2 h with hairpin DNAs. **(C,F,I)** Overnight incubation with hairpin DNAs. Scale bars: 200 μm.

Next, we performed *in situ* HCR for mouse brain using the fluorophore-labeled short hairpin DNAs described above and the reagents and buffers with reaction conditions reported in previous studies (Choi et al., 2014, 2018). Consistent with *in vitro* HCR, 42-nt hairpin DNAs provided stronger *in situ* HCR signals for *Proenkephalin* (*Penk*) mRNA than 36-nt hairpin DNA (Supplementary Figure S3). Thus, 42-nt hairpin DNAs were used for *in situ* HCR to detect mRNAs in this study. *Penk* mRNA-positive cells were abundantly detected in the striatum after HCR amplification initiated by five split-initiator probe sets for *Penk* mRNA (Figure 3). Extending the incubation time with Alexa647-conjugated hairpin pairs (#S41 in Supplementary Table S1) from 45 min to overnight enhanced the fluorescence signal for *Penk* mRNA. The incubation of split-initiator probes with one of the hairpin pairs (H1) or the incubation of hairpin pairs (H1, H2) without split-initiator probes did not generate any signals, indicating the *in situ* HCR specific to *Penk* mRNA (Figure 3). In the absence of initiator probes, background signals slightly increased as the incubation continued.

**Figure 4:**
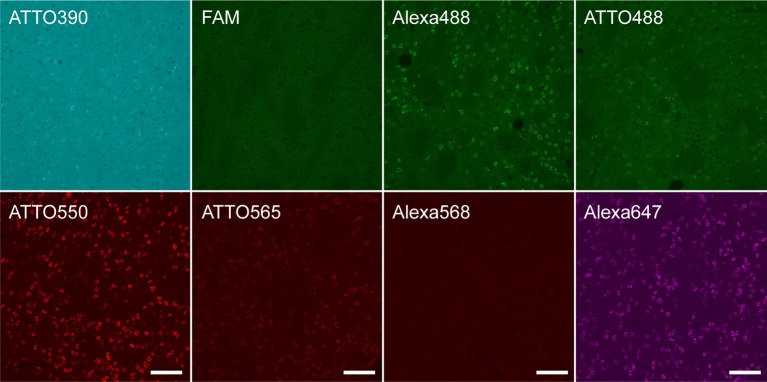
Fluorophores conjugated to hairpin DNAs affect *in situ* HCR signal intensity. *in situ* HCR for *Penk* mRNAs in the mouse striatum using #S41 hairpin DNA. Various fluorophores were conjugated to the hairpin DNAs. All tissue sections were prepared from the same mouse. Background signals were not subtracted. Scale bars: 100 μm.

Since conjugated fluorophores and linkers affected the HCR efficiency in microtubes, we examined the effect of fluorophores and linkers on *in situ* HCR for *Penk* mRNA using the same five probe sets. Although the autofluorescence and detection conditions differed among the color spectra, *Penk*-positive neurons were observed using hairpins labeled with ATTO390, Alexa488, ATTO488, ATTO550, ATTO565, and Alexa647, but the signal intensity of ATTO390, ATTO488 and ATTO565 was weak (Figure 4). We obtained the best and brightest signals with the conjugation of ATTO550, and Alexa647, which is consistent with HCR efficiencies observed in microtubes (Figures 2B,C).

Consistent with *in vitro* HCR (Supplementary Figure S2), ssH-linked ATTO550 produced higher signal intensity for *Penk*-positive neurons than C6-linked ATTO550 (Supplementary Figure S4). The use of the ssH linker for Alexa488 or Alexa647 resulted in slightly stronger signals for *Penk* mRNA than that of the C6 linker (Supplementary Figure S4). PAGE purification of split-initiator probes for *Penk* mRNA increased fluorescent intensity compared with that of unpurified probes (data not shown).

**Figure 5:**
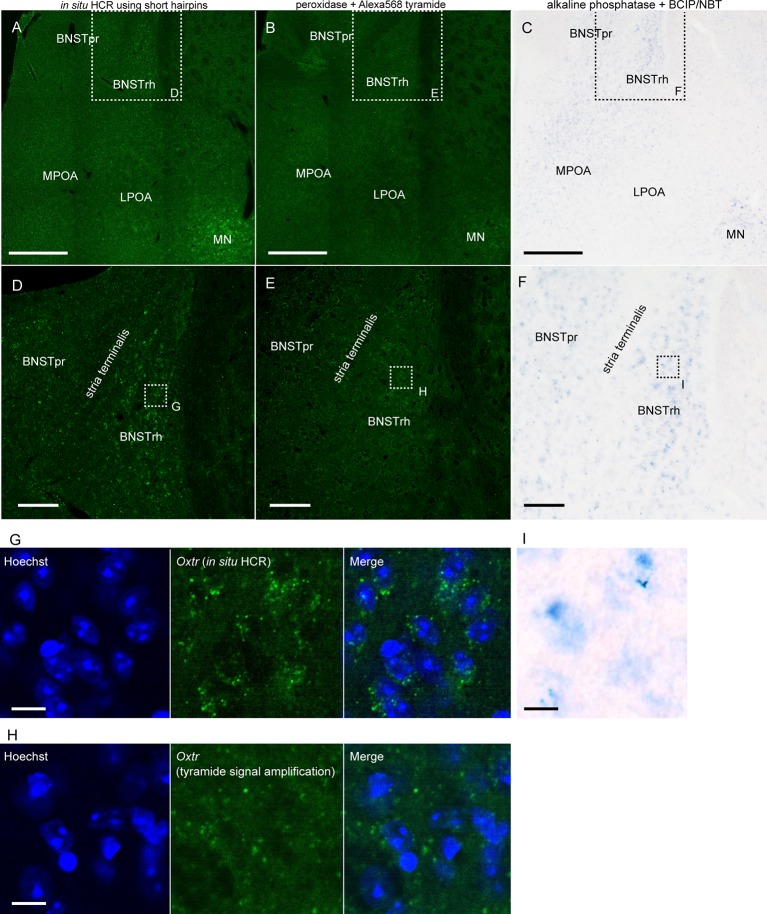
Detection of low abundance mRNA using *in situ* HCR. **(A,D)** *In situ* HCR using ATTO550-conjugated hairpin DNA (#S23) detected *Oxtr* mRNA in mouse brain. Amplification was performed overnight. **(B,E)** Fluorescent ISH with tyramide signal amplification for *Oxtr* mRNA. *Oxtr* mRNA was visualized by a combination of DIG-labeled probe, peroxidase-conjugated anti-DIG antibody, and tyramide Alexa568. **(C,F)** Chromogenic ISH for *Oxtr* mRNA by a combination of DIG-labeled probe, alkaline phosphatase-conjugated anti-DIG antibody, and BCIP/NBT. Color development was performed for 3 days. Panels **(D–F)** corresponds to dashed squares in panels **(A–C)**, respectively. Panels **(G–I)** corresponds to dashed squares in **(D–F)**. Scale bars: 400 μm **(A–C)**, 100 μm **(D–F)** and 10 μm **(G–I)**. BNST, bed nucleus of stria terminalis; BNSTpr, principal nucleus of BNST; BNSTrh, rhomboid nucleus of BNST; LPOA, lateral preoptic area; MN, magnocellular nucleus; MPOA, medial preoptic area.

We compared the sensitivity of *in situ* HCR using short hairpins with other ISH methods by detecting *Oxtr* mRNA, which we selected as an example of very low abundance mRNAs. After hybridization of 10 split-initiator probe sets for *Oxtr* mRNA, *in situ* HCR detected *Oxtr* mRNA signals in the rhomboid nucleus of the bed nucleus of the stria terminalis (BNST) and the magnocellular nucleus (MN) and weak signals in the principal nucleus of the BNST and the medial preoptic area (MPOA; Figures 5A,D; Young et al., 1997; Okabe et al., 2017). ISH with tyramide signal amplification failed to visualize *Oxtr*-positive cells in the BNST (Figures 5B,E). ISH using BCIP/NBT as chromogens demonstrated the distribution of *Oxtr* mRNA-positive cells consistent with that of *in situ* HCR, but there were much weaker and imprecise signals (Figures 5C,F,I). Almost all the HCR signals were observed near cell nuclei (Figure 5G). ISH with tyramide signal amplification produced a low signal-to-noise ratio results in cells at the BNST (Figure 5H).

**Figure 6:**
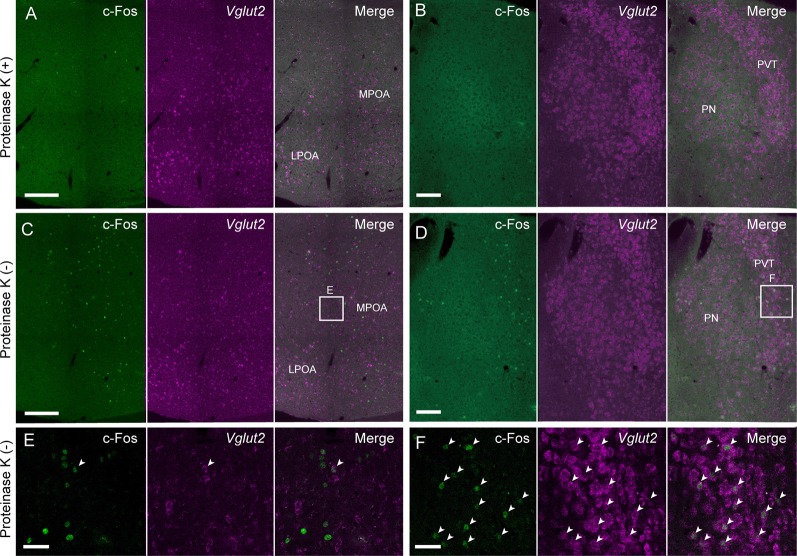
Modified *in situ* HCR without proteinase K treatment. *Vglut2* mRNAs were detected in the mouse medial preoptic area **(A,C,E)** and PVT **(B,D,F)** using Alexa647-conjugated hairpin DNA (#S41). Amplification was performed for 2 h. **(A,B)** Sections were pretreated with proteinase K. **(C,D)** Sections were not treated with proteinase K. Maternal behavior-induced c-Fos immunoreactivity that was diminished by proteinase K treatment before hybridization. Panels **(E,F)** correspond to dashed areas in panels **(C,D)**. Arrowheads indicate double-positive cells of c-Fos protein and *Vglut2* mRNA. LPOA, lateral preoptic area; MPOA, medial preoptic area; PN, parataenial nucleus; PVT, paraventricular thalamus. Scale bars: 200 μm **(A,C)**, 100 μm **(B,D)** and 40 μm **(E,F)**.

Proteinase K treatment has been used for ISH and *in situ* HCR to improve the penetration of RNA/DNA probes and fluorophore-labeled hairpins by digesting proteins. However, proteinase K treatment damages endogenous proteins such as c-Fos, which is a commonly used marker of neural activity, which limits the usefulness of ISH combined with immunohistochemistry. Since our modified *in situ* HCR uses shorter probes than what has been previously used, proteinase K treatment may not be necessary. As predicted, *in situ* HCR using 42-nt hairpins and five sets of 39- and 36-nt split-initiator probes without proteinase K treatment detected *vesicular glutamate transporter* (*Vglut*) *2* in the MPOA (Figure 6C) and the paraventricular thalamus (PVT; Figure 6D) with the same or slightly decreased signal levels as tissues that did undergo proteinase K treatment.

Female mice induced c-Fos expression in the MPOA after the retrieval of pups (Tsuneoka et al., 2013, 2017b; Moffitt et al., 2018). *In situ* HCR without proteinase K treatment successfully detected abundant c-Fos-positive cells in the MPOA and PVT (Figures 6C,D), whereas the number of c-Fos-positive cells and the intensity of c-Fos immunoreactivity were drastically decreased after proteinase K treatment (Figures 6A,B). *Vglut2*-positive neurons were scattered in the MPOA, but most of them were not c-Fos positive (Figures 6E,F).

### Multiplex *in situ* HCR

**Figure 7:**
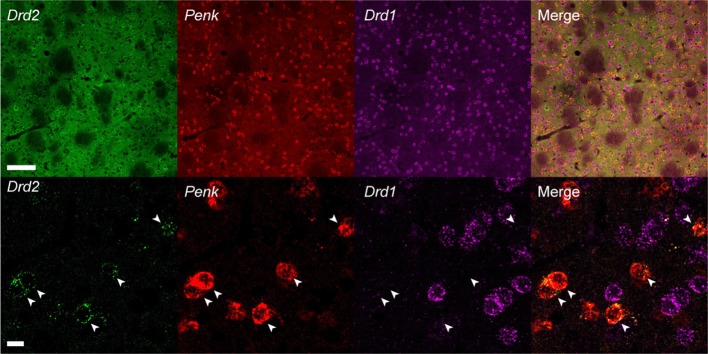
Multiplex *in situ* HCR in the mouse striatum. *Drd2, Penk* and *Drd1* mRNAs were detected by 2-h HCR that simultaneously used different probe-hairpin pairs: Alexa488-conjugated hairpin DNA (#S23) for *Drd2*, ATTO565-conjugated hairpin DNA (#S10) for *Penk*, and Alexa647-conjugated hairpin DNA (#S25) for *Drd1*. In the upper panels, background signals were not subtracted. *Drd2*-positive cells were also *Penk*-positive (arrowheads). Scale bars: 100 μm (Upper panel) and 10 μm (Lower panel).

To test whether the hairpin DNAs separately detect different mRNAs in a single tissue section, we performed multiplexed *in situ* HCR. After 2 h of HCR amplification, *dopamine receptor d1* (*Drd1*), *Drd2* and *Penk* mRNAs were simultaneously detected in the mouse striatum using the corresponding probes (five probe sets for each mRNA) and hairpin DNA pairs conjugated to different fluorophores (Figure 7). Although *Drd1* and *Drd2* mRNA were abundantly expressed in the striatum, double-positive cells for *Drd1* and *Drd2* mRNAs were rarely observed (Figure 7). In contrast, most of the *Drd2*-positive cells were also *Penk*-positive. When observed at higher magnification, the subcellular distribution and abundance of fluorescence for *Drd2* mRNA were distinct from those for *Penk* mRNA.

**Figure 8:**
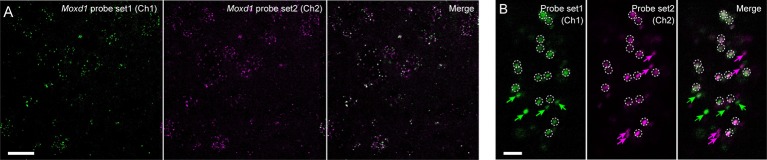
Redundant detection of *Moxd1* mRNA using different probes. **(A)** *In situ* HCR using different probe-hairpin sets designed for different regions of the *Moxd1* mRNAs. HCR for 45 min detected *Moxd1* mRNAs in the bed nucleus of the stria terminalis. Five probe pairs were used for each channel. Fluorescent signals in Ch1 and Ch2 were detected using ATTO550-conjugated hairpin DNA (#S41) and Alexa647-conjugated hairpin DNA (#S25), respectively. **(B)** Representative photomicrograph showing subcellular resolution using the same probe-hairpin sets as in panel **(A)**. Dotted circles indicate granules detected in both channels, and arrows indicate granules detected in one of the two channels. Scale bars: 20 μm **(A)** and 2 μm **(B)**.

We next examined the sensitivity and resolution of modified *in situ* HCR using two different probe sets for *Moxd1* mRNA (Supplementary Table S2), which is a marker of sexually dimorphic nuclei (Tsuneoka et al., 2017a). One of them was five split-initiator probes containing #S41 initiator sequence and detected by ATTO550-conjugated hairpin pairs (#S41) and another was five split-initiator probes containing #S25 initiator sequence and detected by Alexa647-conjugated hairpin pairs (#S25). Both probe-hairpin combinations successfully detected *Moxd1*-positive cells in the BNST by 45-min HCR amplification (Figure 8A), which is consistent with the previous report (Tsuneoka et al., 2017a). At the highest magnification, almost all the ATTO550 and Alexa647 signals were observed as granules that colocalized within the cell (Figure 8B). Whereas 76.9% of ATTO550-positive granules were Alexa647-positive, 74.8% of Alexa647-positive granules were ATTO550-positive (247 and 254 granules in the total count, respectively).

## Discussion

In this study, we designed new short hairpin DNAs and corresponding split initiator probe sets to achieve sensitive detection of various mRNAs. This study proposed short hairpin design rules as well as linker types and fluorophores suitable for *in situ* HCR. These hairpin sets successfully performed HCR both *in vitro* and *in situ* and detected multiple mRNAs simultaneously with virtually the same procedures for a single mRNA. Short probes and hairpin DNAs can penetrate tissues without proteinase K treatment as well as they can tissues with proteinase K treatment. Also, our modified *in situ* HCR technique provided highly sensitive mRNA detection for the visualization of less abundant mRNAs such as *Oxtr*.

### Design of Short Hairpin DNA

To achieve efficient and optimal HCR, the lengths of the total hairpin and each domain are crucial. HCR progresses using the difference in free energy between a long double-strand formed by hairpin pairs and a short double strand of the stem domain of each monomer hairpin. Therefore, longer toehold/loop domains enhance HCR. The HCR efficiency was increased by the extension of the toehold sequence and was independent of the stem sequences shown in Figure 2A. The difference in free energy also depended on the GC contents in the toehold/loop domain. However, increased GC content in the toehold/loop and longer toehold/loop may lead to initiator-independent HCR (Ang and Yung, 2016).

The stability of each hairpin largely depends on the stem length and its sequence. In general, increased stem length and GC content enhance hairpin stability due to an increased number of hydrogen bonds. In this respect, we extended the stem length from 12-nt to 13- or 14-nt by shifting the transition between the stem and loop by one or two nucleotides [e.g., S23-H1: toehold (9 nt) -stem (12 nt)—A—loop (7 nt)—T—stem (12-nt); A and T are transition bases], which often decreased initiator-independent HCR but did not exhibit a decrease in HCR efficiency. Many designed hairpins showed a certain level of initiator-independent HCR, polymerization without an initiator. Many factors cause initiator-independent HCR (Chen et al., 2013). Practically, the purification of synthesized oligos is effective for reducing initiator-independent HCR, although the HPLC purification of DNA hairpins sometimes led to a low HCR efficiency compared with that of PAGE purification (data not shown). We also found that a biased abundance of G or C on one side of the stem domain decreased the stability of the hairpin monomer (Table 1). Although further investigation of the hairpin stability is needed, these data provide useful information regarding the design of new hairpin-based nonenzymatic circuits.

We also found that fluorophores conjugated with hairpin DNAs affect HCR, and the linker length between hairpin DNAs and fluorophores and the structure of fluorophores may be involved in this effect. When a C6-amino linker (6 carbon atom-length) was used to link hairpin DNAs with ATTO550 succinimidyl ester, HCR did not form long products (Supplementary Figure S2). In contrast, the ssH-amino linker (11 atom-length) enabled the HCR using the same hairpin DNA conjugated with ATTO550 (Figure 2 and Supplementary Figure S2), indicating that the use of a longer linker enhances the HCR. Similarly, the signal intensity of ATTO550-labeled hairpin DNAs *via* the ssH linker was brighter than *via* the C6 linker *in situ* (Supplementary Figure S4). Also, the ssH-amino linker achieves a high conjugation efficiency with succinimidyl esters and easy purification (Komatsu et al., 2008). We found efficient HCR amplification with fluorophore-labeled hairpin DNAs conjugated through an ssH-amino linker.

The presence of a linker-like linear structure in fluorophores is an additional factor to be considered for higher signal gain. Among the fluorophores examined, Alexa647, ATTO550, and ATTO488 have a linker-like linear structure (Figure 2). Hairpin DNAs conjugated with Alexa647 using the C6-amino linker led to over 10 kb HCR products (Supplementary Figure S2). Many fluorophores, such as the Alexa and ATTO series, have a comparable molecular structure and steric size to a single nucleotide molecule. Therefore, the use of a long linker and fluorophore with a linker-like linear structure is preferred because it is unlikely to cause steric hindrance against forming double-strands between hairpin DNA pairs. We found efficient HCR amplification with fluorophore-labeled hairpin DNAs conjugated through an ssH-amino linker, except ATTO565 and Alexa568 (Figure 2B), which have a very large molecular structure without any linker-like structure, although Alexa568 is widely used bright fluorophore in the immunohistochemistry and ISH. Such steric hindrance was further suggested in performing *in situ* HCR (Figure 4). The molecular structures of ATTO550 and Alexa647 succinimidyl esters are also very large, but they have a linker-like structure near the binding site, enabling efficient HCR amplification.

### *In situ* HCR Using Short Hairpin DNA

The signal gain of HCR amplification by short hairpin DNAs was also confirmed in the mouse brain. *In situ* HCR showed that *Penk* mRNA was uniformly expressed in the mouse striatum (Figure 3), which was consistent with the results from previous ISH studies (Turchan et al., 1997; Lobo et al., 2006; Labouesse et al., 2018). The signal for *Penk* developed intensely after an overnight incubation as HCR amplification proceeded, although the overnight incubation also resulted in slight background staining. Such *Penk*-positive cells were never observed in the reaction buffer without H2 hairpin DNA, suggesting that simple hybridization of H1 with a probe for *Penk* mRNA was not enough to visualize *Penk* mRNA-positive cells.

Although modification and adoption of the short hairpin *in situ* HCR was reported to detect very abundant neuropeptide mRNAs (Sui et al., 2016), the amplification reached a plateau within an hour and the polymerized products were much smaller (approximately 1 kb) than those observed in the current study.

The gel electrophoresis of our short hairpin DNAs showed at least 400-fold amplification to develop more than 10 kb double-strand products from 42 nt single-strand hairpin DNAs (Figure 2). Compared with 42-nt hairpin DNAs, 36-nt hairpin DNAs exhibited lower HCR efficiency *in vitro* (Figure 2A) and *in situ* (Supplementary Figure S3), which may be due to a short toehold/loop length that renders hairpin monomer stable.

Such strong amplification by HCR using our short hairpin DNAs enabled us to visualize low abundance mRNAs such as *Oxtr* mRNA. Although G-protein-coupled receptors are often difficult to visualize by enzyme-based fluorescent ISH because of their low abundance, *in situ* HCR successfully detected weak *Oxtr* mRNA expression in the MPOA and BNST, consistent with previous studies (Young et al., 1997; Okabe et al., 2017) and Allen brain atlas^3^. *Oxtr* mRNA was detected using chromogenic ISH (CISH) using alkaline phosphatase and BCIP/NBT after a long chromogenic reaction which is thought to be most sensitive among enzyme-based conventional ISH (Bonn et al., 2012). We also confirmed that the sensitivity of CISH was superior to that of ISH with tyramide signal amplification for low abundance mRNA. Importantly, the distribution of *Oxtr* mRNA detected by *in situ* HCR was largely matched with the result of CISH, suggesting that the sensitivity of *in situ* HCR was comparable to that of CISH. Furthermore, *in situ* HCR has several advantages over CISH such as simultaneous multiplex imaging and an elevated ability for the probes to penetrate.

### Probe Design

In this study, we detected six genes using five or ten sets of split probes per gene. In the original report of split probes (Choi et al., 2018), the authors recommended more than 20 sets of probes to increase signal/noise ratio and precision, because the number of probes theoretically correlates signal intensity. As shown in Figure 8, five sets of probes can visualize a single mRNA, although the imaging of a single-molecule requires a high N.A. lens (e.g., >1.3). Ten sets of probes allowed us to visualize low abundance mRNA such as *Oxtr* at a relatively low magnification using a 20× objective lens with 0.75 N.A. (Figure 5). Additional probe sets may increase signal/noise ratio and precision, while it costs more. The minimum number of probes for appropriate imaging needs to be determined by users based on many factors including the imaging environment, the intensity of autofluorescence, conjugated fluorophore, required resolution, and precision.

We designed split probes according to Choi et al. (2018). The sequence for probes were chosen in consideration of GC contents and homology without further optimization and evaluation, because the split probes were very robust for non-specific hybridization (Choi et al., 2018). Gene expressions shown in this study were consistent with previous studies. Therefore, the design of new probes for target genes is as easy and fast as probes for conventional ISH.

Probe penetration is a key factor for the sensitivity of ISH. To enhance penetration, proteinase K treatment has been widely used in ISH in addition to the fragmentation of long probes, although some endogenous proteins are degraded by proteinase K treatment. Some reports proposed organic solvents as an alternative to proteinase K treatment in the *Drosophila* embryo (Nagaso et al., 2001; Jaeger et al., 2004), but these techniques were not as effective as the proteinase K treatment in mouse neural tissue (data not shown). Therefore, in the case of ISH with immunohistochemistry, there was a trade-off in the use of proteinase K between better ISH probe penetration and better preservation of antigens, such as c-Fos, for immunohistochemistry of mouse neural tissue. There was a report on the combined use of *in situ* HCR with immunohistochemistry to detect both mRNA and proteins (Zhuang et al., 2020). However, the authors adopted proteinase K treatment, which requires additional optimization of immunohistochemistry such as adjusting the concentration of antibodies. *In situ* HCR using short hairpins overcomes these issues. *In situ* HCR without proteinase K treatment showed *Vglut2* mRNA signals comparable to those observed following proteinase K treatment (Figure 6).

### Multiplex *in situ* HCR

Using three hairpin DNA sets, *in situ* HCR demonstrated that *Penk*-positive cells in the mouse striatum were also *Drd2*-positive, but *Drd1*-negative (Figure 7). Almost all striatal neurons in mice express either *Drd1* or *Drd2* mRNA, and *Drd2*-positive neurons express *Penk* mRNA, as demonstrated by ISH, cell-assembly microarray using transgenic reporter mice and single-cell RNA-seq (Lobo et al., 2006; Heiman et al., 2008; Gokce et al., 2016; Labouesse et al., 2018). *In situ* HCR in this study exactly reproduced cell data revealed by the previous studies. Thus, multiplex *in situ* HCR works to visualize different sets of mRNAs simultaneously with a high signal-to-noise ratio. Tyramide signal amplification has been a popular approach for performing multiplexed fluorescent ISH (Zaidi et al., 2000; Lauter et al., 2011a,b; Bonn et al., 2012; Tsuneoka et al., 2015, 2017b). It has been used with different hapten-labeled probes, peroxidase-conjugated anti-hapten antibodies, and fluorophore-conjugated tyramides. ISH using tyramide signal amplification requires optimization for each step, requiring substantial labor from researchers. Also, antibody reactions and subsequent amplification steps should be performed separately for each target mRNA after the deactivation of the enzyme. In contrast, the *in situ* HCR system can simultaneously develop multiple colors and show autonomous suppression of noise without further optimization per target mRNA (Choi et al., 2014, 2018). Such advantages were also observed with the short hairpin DNAs designed in this study. Moreover, the current modification for the short hairpin DNA design implies further signal amplification by modifying the hairpin sequence, such as is used in the branched HCR system (Xu and Zheng, 2016; Bi et al., 2017; Liu et al., 2018; Wu et al., 2019).

Single-molecule fluorescent ISH has become a powerful technique not only for analyzing subcellular localization of specific mRNAs (Chen et al., 2016; Samacoits et al., 2018) but also for demonstrating the presence of low abundance mutant mRNAs (Haimovich and Gerst, 2018; Marras et al., 2019), long noncoding RNAs (Chen et al., 2016), ribosome-mRNA interactions (Burke et al., 2017) and comprehensive transcriptional analyses *in situ* (Shah et al., 2016; Moffitt et al., 2018). The signals from two different probes designed for detection of *Moxd1* mRNA were largely matched to each other at subcellular resolution (Figure 8). This suggests that *in situ* HCR using short hairpin DNAs is also applicable for single-molecule fluorescent ISH with a specific imaging device, similar to the original hairpin *in situ* HCR methods (Choi et al., 2014, 2018). Because, we used a small number of probe sets (five probe sets) and the thresholds were determined to minimize false-positive signals derived from tissue autofluorescence, not all the signal from one channel coincide with those from another channel. Increasing the number of probe sets will gain a higher signal/noise ratio and precision.

### Summary and Advantage of Short Hairpin DNA

In summary, our short hairpin *in situ* HCR enables the visualization of low abundance mRNA and multiple mRNAs and the simultaneous detection of mRNA and protein without proteinase K treatment. When designing short hairpins, the following points should be taken into account; length of toehold/loop, end bases of toehold/loop, the number of G and C on one strand of the stem domain, structures of fluorophores and linker length. The advantages of short hairpin DNAs are low cost and high permeability without proteinase K treatment. Given that the length of DNA decreases from 72 nt to 42 nt, the cost for oligo DNA synthesis per mole decreases by 66% (1–42^2^/72^2^). Also, as the oligos get longer, mis-synthesized oligos increase exponentially, which further makes short hairpins cost-effective.

## Data Availability Statement

All data are available from the corresponding authors upon reasonable request.

## Ethics Statement

The animal study was reviewed and approved by Institutional Animal Care and Use Committee of Toho University.

## Author Contributions

YT: conceptualization, methodology and formal analysis. YT and HF: writing, funding acquisition and supervision.

## Conflict of Interest

The authors declare that the research was conducted in the absence of any commercial or financial relationships that could be construed as a potential conflict of interest.

## Footnotes

^1^https://blast.ncbi.nlm.nih.gov

^2^http://www.nupack.org/

^3^http://mouse.brain-map.org
