# Supplementary material for: Modified in situ Hybridization Chain Reaction Using Short Hairpin DNAs
Source: Front Mol Neurosci. 2020 May 12;13:75. doi: 10.3389/fnmol.2020.00075 (PMC7235299; doi:10.3389/fnmol.2020.00075)
Supplement: Supplementary file 1 [file Data_Sheet_1.PDF]

Supplementary Material

Supplementary Figures and Tables

Supplementary Figures

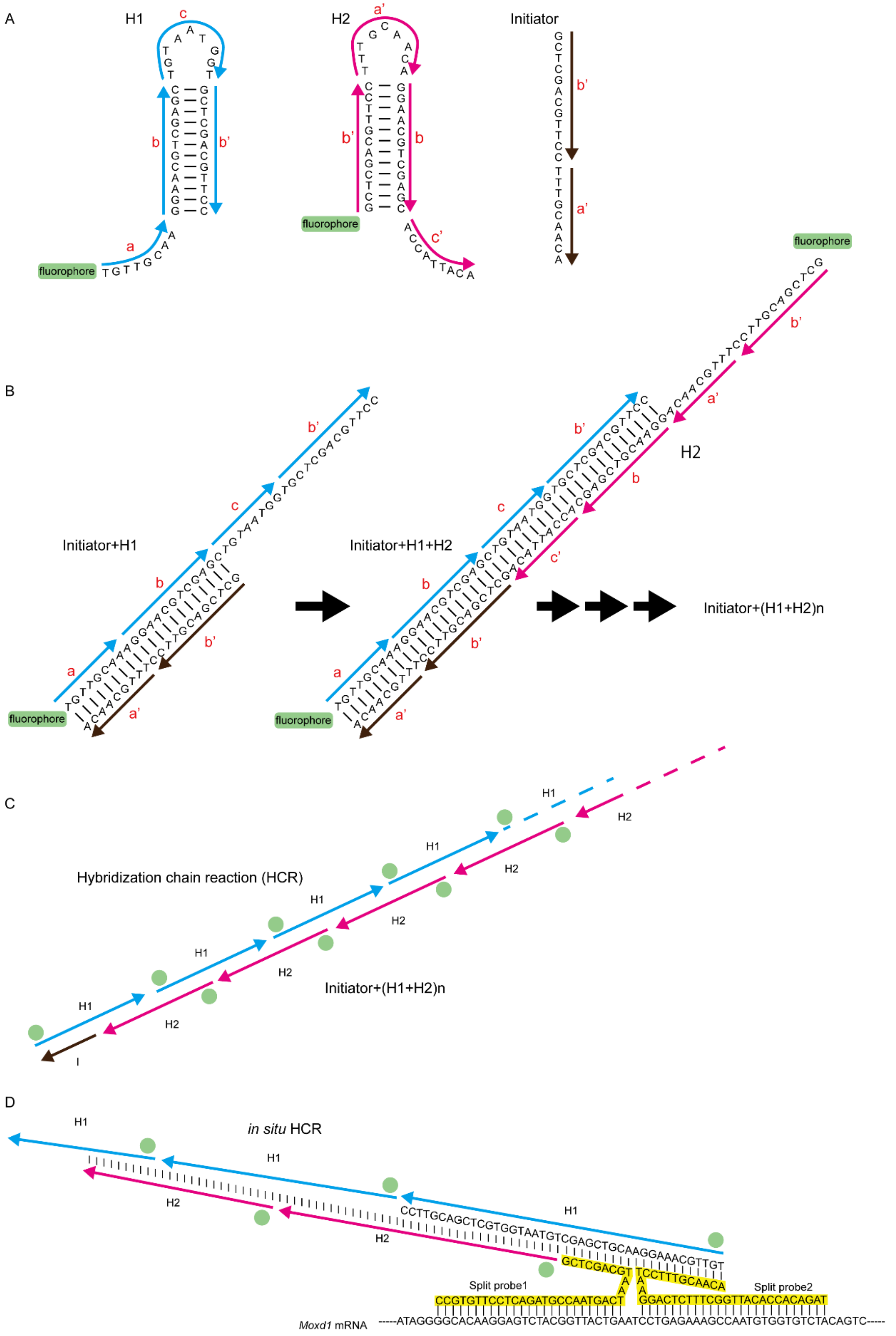

Supplementary Figure 1. Modified short hairpin for *in situ* HCR.

A: Sequence and structure of H1 and H2 of hairpin DNA pairs (#41) and corresponding initiator sequence. B: The initiator hybridizes with the toehold and stem domains (a, b) of H1. Then, the initiator, H1 complex hybridizes with the toehold and stem domains (c’, b) of H2. C: H1 and H2 continue to hybridizes with each other. D: Split-initiator probes (yellow) have a sequence hybridized to *Moxd1* mRNA, half of the initiator sequence, and 2 nt spacer sequence. Binding of the split probe set to *Moxd1* mRNA triggers HCR of fluorophore-labelled hairpin DNAs, H1 and H2.

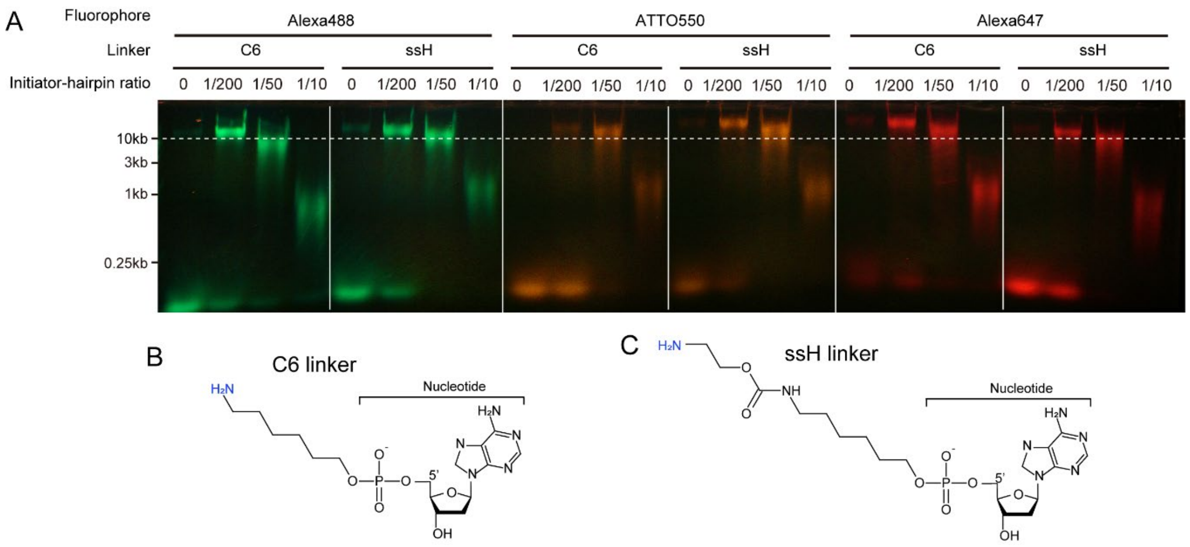

Supplementary Figure 2. Effect of linkers on HCR.

(A) All reaction mixtures contained 0.5 µM hairpin DNAs and four concentrations of initiator DNAs (0, 0.0025, 0.01, 0.05 µM) and the HCR products were visualized after agarose gel electrophoresis. C6 or ssH linkers were used to conjugate fluorophores with #S41 Hairpin DNAs. Captured images taken with appropriate filter sets were overlaid based on the loading position. (B, C) C6 linker (B) or ssH linker (C) conjugated with a nucleotide. The leftmost amino group (blue) is to conjugate with fluorophore. After bonding reaction, the amine of the linker and N-hydroxysuccinimide of a fluorophore (Figure 2D-2K) is replaced to the amide bond.

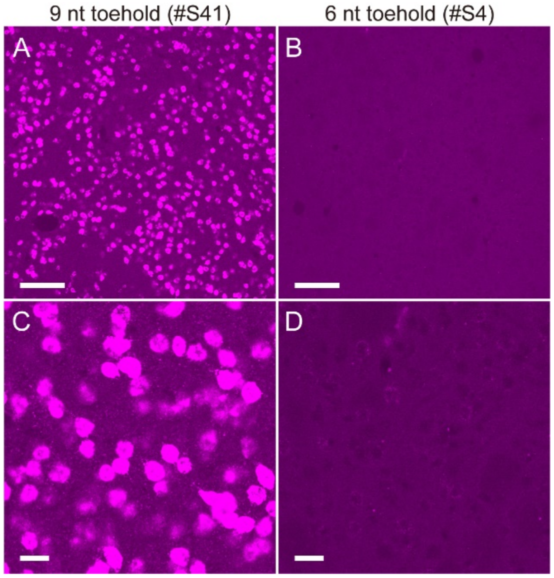

Supplementary Figure 3. Toehold length of the short hairpins affects *in situ* HCR intensity

In situ HCR for *Penk* mRNAs in the mouse striatum using #S41 (A, C) and #S4 (B, D) hairpin DNA conjugated with Alexa647. Tissue sections were prepared from the same mouse. Photomicrograph of A is the same as Supplementary Figure 4E. C and D are high magnification images. Background signals were not subtracted. Scale bars: 100 (A, B) and 20 (C, D) µm.

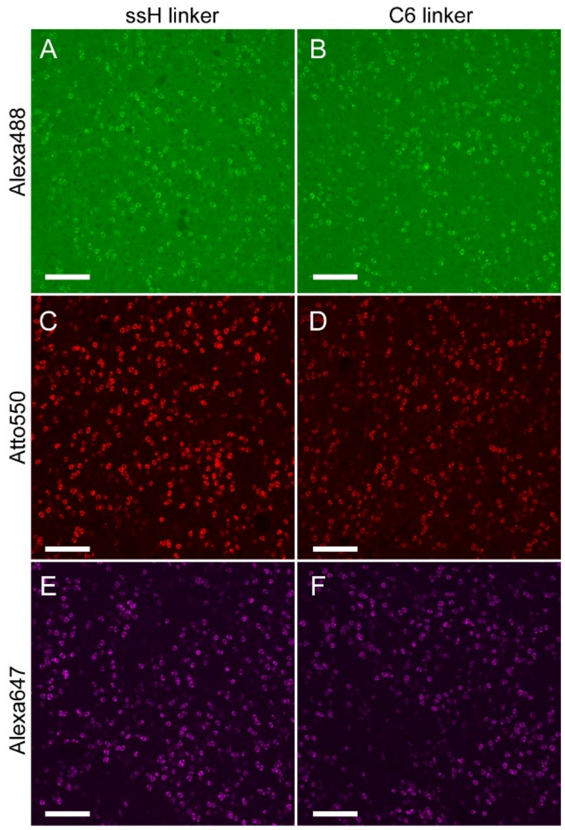

Supplementary Figure 4. Linker length conjugated to hairpin DNA and *in situ* HCR intensity

In situ HCR for *Penk* mRNAs in the mouse striatum using S41 hairpin DNA. (A, C, E) ssH linker. (B, D, F) C6 linker. A, B: Alexa488. C, D: Atto550. E, F: Alexa647. All tissue sections were prepared from the same mouse. Background signals were not subtracted. Scale bars: 100 µm.

frontiers

Supplementary Table 1. Summary of short hairpin and initiator sets and their HCR.

Hairpin ID		Sequence (stem sequences are underlined)	Length (nt)	*Initiator-independent HCR	HCR efficiency	
					**Unreacted hairpin band	HCR product size
S1	H1	TCCAATCGATCGGCCAGCTGAGATGCTGGCCGATCG	36	Faint	+	10kb
	H2	GCTGGCCGATCGATTGGACGATCGGCCAGCATCTCA	36			
	Initiator	GCTGGCCGATCGATTGGA	18			
S2	H1	CATGATCTAACTCTCCCCAACAGAGGGGAGAGTTAG	36	Abundant	+	10kb
	H2	GGGGAGAGTTAGATCATGCTAACTCTCCCCTCTGTT	36			
	Initiator	GGGGAGAGTTAGATCATG	18			
S3	H1	TAATGGGCTCATCCGACGACGCGACGTCGGATGAGC	36	Mild	+	10kb
	H2	CGTCGGATGAGCCCATTAGCTCATCCGACGTCGCGT	36			
	Initiator	CGTCGGATGAGCCCATTA	18			
S4	H1	TGCAAAGGAACGTCGAGCAATGGTGCTCGACGTTCC	36	Not recognized	+	10kb
	H2	GCTCGACGTTCCTTTGCAGGAACGTCGAGCACCATT	36			
	Initiator	GCTCGACGTTCCTTTGCA	18			
S5	H1	CATGTACTGACGCTCCACAACAGAGTGGAGCGTCAG	36	Faint	+	10kb
	H2	GTGGAGCGTCAGTACATGCTGACGCTCCACTCTGTT	36			
	Initiator	GTGGAGCGTCAGTACATG	18			
S6	H1	TAATGGGCTCATCCGACGACTTGACGTCGGATGAGC	36	Not recognized	+	10kb
	H2	CGTCGGATGAGCCCATTAGCTCATCCGACGTCAAGT	36			
	Initiator	CGTCGGATGAGCCCATTA	18			
S7	H1	TCCAAAGGAACGTCGAGCTGAGATGCTCGACGTTCC	36	Not recognized	-	<10kb
	H2	GCTCGACGTTCCTTTGGAGGAACGTCGAGCATCTCA	36			
	Initiator	GCTCGACGTTCCTTTGGA	18			
S8	H1	GTTGCAAAGGAACGTCGAGCGTAATGGTGCTCGACGTTCC	40	Not recognized	+	10kb
	H2	GCTCGACGTTCCTTTGCAACGGAACGTCGAGCACCATTAC	40			
	Initiator	GCTCGACGTTCCTTTGCAAC	20			

S9	H1	TTGTTGCAAAGGAACGTCGAGCTTGTAA TGGTGCTCGACGTTCC	44	Not recognized	-	10kb
	H2	GCTCGACGTTCCTTTGCAACATGGAACG TCGAGCACCATTACAA	44			
	Initiator	GCTCGACGTTCCTTTGCAACAT	22			
S10	H1	ATCTAATGGGCTCATCCGACGATCACTT GACGTCGGATGAGC	42	Faint	-	10kb
	H2	CGTCGGATGAGCCCATTAGATGCTCATC CGACGTCAAGTGAT	42			
	Initiator	CGTCGGATGAGCCCATTAGAT	21			
S11	H1	TGTTTATGTCGTGGTCACCGCAATATGCC AGCGGTGACCACG	42	Mild	+	10kb
	H2	GCGGTGACCACGACATAAACACGTGGTC ACCGCTGGCATATT	42			
	Initiator	GCGGTGACCACGACATAAACA	21			
S12	H1	GATTAAGGTGCGTGTACAGCGAAACGCA AACGCTGTACACGC	42	Mild	-	10kb
	H2	CGCTGTACACGCACCTTAATCGCGTGTA CAGCGTTTGCGTTT	42			
	Initiator	CGCTGTACACGCACCTTAATC	21			
S13	H1	TAAAGTGAGCGCACTGCGACGTTCTGCT TTCGTCGCAGTGCG	42	Abundant	-	10kb
	H2	CGTCGCAGTGCGCTCACTTTACGCACTGC GACGAAAGCAGAA	42			
	Initiator	CGTCGCAGTGCGCTCACTTTA	21			
S14	H1	TGTGTATGTCGTGGTCCTCGCAAGTATCC AGCGAGGACCACG	42	Abundant	-	10kb
	H2	GCGAGGACCACGACATACACACGTGGTC CTCGCTGGATACTT	42			
	Initiator	GCGAGGACCACGACATACACA	21			
S15	H1	AGTTGAAGTGCGTGTACAGGGAAGACCA AACCCTGTACACGC	42	Mild	-	10kb
	H2	CCCTGTACACGCACTTCAACTGCGTGTAC AGGGTTTGGTCTT	42			
	Initiator	CCCTGTACACGCACTTCAACT	21			
S16	H1	TTGAAGTTGCGATCCCGTGCCACAAGAA CAGGCACGGGATCG	42	Abundant	-	10kb
	H2	GGCACGGGATCGCAACTTCAACGATCCC GTGCCTGTTCTTGT	42			
	Initiator	GGCACGGGATCGCAACTTCAA	21			
S17	H1	TTACGACTTCGACGACCACCCTACTTGA ATGGGTGGTCGTCG	42	Faint	-	10kb
	H2	GGGTGGTCGTCGAAGTCGTAACGACGAC CACCCATTCAAGTA	42			
	Initiator	GGGTGGTCGTCGAAGTCGTAA	21			
S18	H1	TAGAATGAGCCCACTCCGACGTTCTGTCT TCGTCGGAGTGGG	42	Mild	-	10kb
	H2	CGTCGGAGTGGGCTCATTCTACCCACTCC GACGAAGACAGAA	42			
	Initiator	CGTCGGAGTGGGCTCATTCTA	21			

S19	H1	AGTACATGTCGTGGTCCTAGCTCATATGCAGCTAGGACCACG	42	Mild	-	10kb
	H2	GCTAGGACCACGACATGTACTCGTGGTCCTAGCTGCATATGA	42			
	Initiator	GCTAGGACCACGACATGTACT	21			
S20	H1	AGTTGAAGTGCTCGAACCTGGAAGACCAAACCAGGTTCGAGC	42	Mild	-	10kb
	H2	CCAGGTTCGAGCACTTCAACTGCTCGAACCTGGTTTGGTCTT	42			
	Initiator	CCAGGTTCGAGCACTTCAACT	21			
S21	H1	TTGAAGTTGCGACCTCGTACCACAAGAACAGGTACGAGGTCG	42	Abundant	-	10kb
	H2	GGTACGAGGTCGCAACTTCAACGACCTCGTACCTGTTCTTGT	42			
	Initiator	GGTACGAGGTCGCAACTTCAA	21			
S22	H1	TTACGACTTCGACGACCATCCTACTTGAATGGATGGTCGTCG	42	Abundant	±	10kb
	H2	GGATGGTCGTCGAAGTCGTAACGACGACCATCCATTCAAGTA	42			
	Initiator	GGATGGTCGTCGAAGTCGTAA	21			
S23	H1	ATACGACTTCGACGACCACCCAACTTGAATGGGTGGTCGTCG	42	Faint	-	10kb
	H2	GGGTGGTCGTCGAAGTCGTATCGACGACCACCCATTCAAGTT	42			
	Initiator	GGGTGGTCGTCGAAGTCGTAT	21			
S24	H1	TAGAATGAGCCAACTCCGACGTTCTGTCTTCGTCGGAGTTGG	42	Faint	+	10kb
	H2	CGTCGGAGTTGGCTCATTCTACCAACTCCGACGAAGACAGAA	42			
	Initiator	CGTCGGAGTTGGCTCATTCTA	21			
S25	H1	TAGACTGAACCCACTCCGACGATCTGTCTTCGTCGGAGTGGG	42	Not recognized	-	10kb
	H2	CGTCGGAGTGGGTTCAGTCTACCCACTCCGACGAAGACAGAT	42			
	Initiator	CGTCGGAGTGGGTTCAGTCTA	21			
S26	H1	TGACATGTACTGACGCTCCACAGTAACAGAGTGGAGCGTCAG	42	Mild	+	10kb
	H2	GTGGAGCGTCAGTACATGTCACTGACGCTCCACTCTGTTACT	42			
	Initiator	GTGGAGCGTCAGTACATGTCA	21			
S27	H1	ATCTAAGATGCTCACCTGACGTTCATGTAACGTCAGGTGAGC	42	Not recognized	+	10kb
	H2	CGTCAGGTGAGCATCTTAGATGCTCACCTGACGTTACATGAA	42			
	Initiator	CGTCAGGTGAGCATCTTAGAT	21			
S28	H1	AGTACATGTCGTGGTCCTAGCTTGTATGAAGCTAGGACCACG	42	Abundant	-	10kb
	H2	GCTAGGACCACGACATGTACTCGTGGTCCTAGCTTCATACAA	42			
	Initiator	GCTAGGACCACGACATGTACT	21			

S29	H1	AGTTGAAGTGCTCGGACCTGGAAGACTA ATCCAGGTCCGAGC	42	Mild	-	10kb
	H2	CCAGGTCCGAGCACTTCAACTGCTCGGA CCTGGATTAGTCTT	42			
	Initiator	CCAGGTCCGAGCACTTCAACT	21			
S30	H1	ATGAAGTATCGACCTCGTACCAGAAGAA CTGGTACGAGGTCG	42	Faint	-	10kb
	H2	GGTACGAGGTCGATACTTCATCGACCTC GTACCAGTTCTTCT	42			
	Initiator	GGTACGAGGTCGATACTTCAT	21			
S31	H1	TAACATGTACTGACGCTCCACTCTAACA GAGTGGAGCGTCAG	42	Not recognized	+	10kb
	H2	GTGGAGCGTCAGTACATGTTACTGACGC TCCACTCTGTTAGA	42			
	Initiator	GTGGAGCGTCAGTACATGTTA	21			
S32	H1	AAATCCAATCGATCGGCCAGCAATTGAG ATGCTGGCCGATCG	42	Mild	-	10kb
	H2	GCTGGCCGATCGATTGGATTTCGATCGG CCAGCATCTCAATT	42			
	Initiator	GCTGGCCGATCGATTGGATTT	21			
S33	H1	ATGTAAGATGCTCACCTGACGTTCATGT AACGTCAGGTGAGC	42	Not recognized	-	10kb
	H2	CGTCAGGTGAGCATCTTACATGCTCACCT GACGTTACATGAA	42			
	Initiator	CGTCAGGTGAGCATCTTACAT	21			
S34	H1	ATGGAAGATGCTCACCGACCGTTCATGC AACGGTCGGTGAGC	42	Not recognized	-	10kb
	H2	CGGTCGGTGAGCATCTTCCATGCTCACC GACCGTTGCATGAA	42			
	Initiator	CGGTCGGTGAGCATCTTCCAT	21			
S35	H1	AGTACATGTCGTGGTGGTAGCTTGTATG AAGCTACCACCACG	42	Not recognized	-	10kb
	H2	GCTACCACCACGACATGTACTCGTGGTG GTAGCTTCATACAA	42			
	Initiator	GCTACCACCACGACATGTACT	21			
S36	H1	ACATGAAGTGCTCGGAGGTGGATGACTA ATCCACCTCCGAGC	42	Not recognized	+	<10k b
	H2	CCACCTCCGAGCACTTCATGTGCTCGGA GGTGGATTAGTCAT	42			
	Initiator	CCACCTCCGAGCACTTCATGT	21			
S37	H1	ATGAAGTATCGACCTCGTCCCAGAAGAA CTGGGACGAGGTCG	42	Faint	-	10kb
	H2	GGGACGAGGTCGATACTTCATCGACCTC GTCCCAGTTCTTCT	42			
	Initiator	GGGACGAGGTCGATACTTCAT	21			
S38	H1	TAGCATGTACTGACGCTCCACTTCAACC AAGTGGAGCGTCAG	42	Faint	-	10kb
	H2	GTGGAGCGTCAGTACATGCTACTGACGC TCCACTTGGTTGAA	42			
	Initiator	GTGGAGCGTCAGTACATGCTA	21			

S39	H1	AAATCCAAT<u>CCACCGACCAGC</u>AATTGAGAT<u>GCTGGTCGGTGG</u>	42	Faint	-	10kb
	H2	<u>GCTGGTCGGTGG</u>ATTGGATTT<u>CCACCGACCAGC</u>ATCTCAATT	42			
	Initiator	GCTGGTCGGTGGATTGGATTT	21			
S40	H1	TGAACCTTA<u>GGTCGTAGGAGC</u>ACTTTCACT<u>GCTCCTACGACC</u>	42	Faint	-	10kb
	H2	<u>GCTCCTACGACC</u>TAAGGTTCA<u>GGTCGTAGGAGC</u>AGTGAAAGT	42			
	Initiator	GCTCCTACGACCTAAGGTTCA	21			
S41	H1	TGTTGCAAA<u>GGAACGTCGAGC</u>TGTAATGGT<u>GCTCGACGTTCC</u>	42	Not recognized	-	10kb
	H2	<u>GCTCGACGTTCC</u>TTTGCAACA<u>GGAACGTCGAGC</u>ACCATTACA	42			
	Initiator	GCTCGACGTTCCTTTGCAACA	21			

Note:

*Initiator-independent HCR

Mild: the abundance of initiator-independent HCR was less than that of unreacted hairpins.
Abundant: the abundance of initiator-independent HCR was same or more than that of unreacted hairpins.

**HCR efficiency

+: Unreacted hairpin band was observed after HCR with an initiator/short hairpin concentration ratio of 1/50.

-: No hairpin band was observed after HCR with an initiator/short hairpin concentration ratio of 1/50.

Supplementary Table 2. Split-initiator probe sequences.

Probe Name*	First probe				Second probe		
	25nt, rev-comp to target mRNA		3nt of b'	H2 loop	latter 9nt of b'		25nt, rev-comp to target mRNA
Drd1-1S25	GGGATGCTGCCTCTTCTTCTGAGAC	AA	GGG	TTCAGTCTA	CGTCGGAGT	AA	AGCCTAAAATACATGCATTTCTCCT
Drd1-2S25	TACCAGGAAGAAGAGCCGCTTGCTT	AA	GGG	TTCAGTCTA	CGTCGGAGT	AA	CACCTGTCTTCTGGGTTCAGTGCTC
Drd1-3S25	GAGTGGACAGGATAAGCAGGGACAG	AA	GGG	TTCAGTCTA	CGTCGGAGT	AA	AACAGGCTGTGAGGATGCGAAAGGA
Drd1-4S25	GAACCCAATATTCAGGTTGAATGCT	AA	GGG	TTCAGTCTA	CGTCGGAGT	AA	CCGCTGTGGGTAACGGGTTGGATCT
Drd1-5S25	GCCTTCCCAGAAGTCATTCCCAGCT	AA	GGG	TTCAGTCTA	CGTCGGAGT	AA	GAATCTCAGAGTCTATGTGTACTGT
Drd2-1S23	TCTGCCTCTCCAGATCATCATCGTA	AA	TCG	AAGTCGTAT	GGGTGGTCG	AA	AGGACAGGTTCAGTGGATCCATTGG
Drd2-2S23	AGTTTCATGTCCTCAGGGTGGGTAC	AA	TCG	AAGTCGTAT	GGGTGGTCG	AA	TTGCCCTTGAGTGGTGTCTTCAGGT
Drd2-3S23	GTGGGATGTTGCAGTCACAGTGTAT	AA	TCG	AAGTCGTAT	GGGTGGTCG	AA	TCAGGATGTGCGTGATGAAGAAGGG
Drd2-4S23	TCTTCATGAAGGCCTTGCGGAACTC	AA	TCG	AAGTCGTAT	GGGTGGTCG	AA	TGTTGAAGGTGGTATAGATGATGGG
Drd2-5S23	GGAAGGCTGCTGCTTCTTTGGTGCC	AA	TCG	AAGTCGTAT	GGGTGGTCG	AA	GGAGACAGGATCTGCATGTGAAAGG
Moxd1-1S25	CTTGTCATTGACATCGCACGTGTGC	AA	GGG	TTCAGTCTA	CGTCGGAGT	AA	CTCCCTGCTAAACTCGATCACTGTG
Moxd1-2S25	GGACCAGGTTCTCATGGCCTCTCTC	AA	GGG	TTCAGTCTA	CGTCGGAGT	AA	TTATTGGCTCCACCTTTATCACATG
Moxd1-3S25	CCTCCTCTAGGCACTCCAGAGTGCA	AA	GGG	TTCAGTCTA	CGTCGGAGT	AA	GACCCTCAGAATGGAACTCGGGCAT
Moxd1-4S25	TGCTTTCTCGCACACCAGAGGTTCT	AA	GGG	TTCAGTCTA	CGTCGGAGT	AA	TTCATAAGGCCTTTTGATATCAGGA
Moxd1-5S25	GAGGAAGCCAAAGATGCAGAGATAA	AA	GGG	TTCAGTCTA	CGTCGGAGT	AA	ACTGAGGTCAGAGGACCATGAGTAA
Moxd1-6S41	TAGACACCACATTGGCTTTCTCAGG	AA	TCC	TTTGCAACA	GCTCGACGT	AA	TCAGTAACCGTAGACTCCTTGTGCC
Moxd1-7S41	AAAGCACATAACGAGGATCCAGTGG	AA	TCC	TTTGCAACA	GCTCGACGT	AA	TGCCGAGGGATAAGCCAACGTGAGG
Moxd1-8S41	TCACATTCACAGGCAGGCTAAGAAC	AA	TCC	TTTGCAACA	GCTCGACGT	AA	GCTTGTTGAAGGACAGCCCTTCCTT
Moxd1-9S41	GAGTATGACAGAAGTCACAAGCCCT	AA	TCC	TTTGCAACA	GCTCGACGT	AA	CTGCTCAGCATACTTCCAAGAAGCA
Moxd1-10S41	ACCCAGAAAGGATGAAGAGCAAATG	AA	TCC	TTTGCAACA	GCTCGACGT	AA	ATCGGAGAGTAAGTCCACTAGTGTT
Penk-1S41	AGTGATGCCTGGGACTATTCTATCT	AA	TCC	TTTGCAACA	GCTCGACGT	AA	CTGGAGGTATCCTATCTTCCCACGG
Penk-2S41	ATATAGCTCGTCCATCTTCTTCATG	AA	TCC	TTTGCAACA	GCTCGACGT	AA	GCCTCCGTACCGTTTCATGAAGCCT
Penk-3S41	AGTTGGCCAAGGTGTCTCCCTCATC	AA	TCC	TTTGCAACA	GCTCGACGT	AA	CATCCTTCTTCATGAAGCCGCCATA

Penk-4S41	CATACCTCTTGCTCATGTCTTCGTC	AA	TCC	TTTGCAACA	GCTCGACGT	AA	TGTTGGTGCTCTCTTGTTGGTGGCT
Penk-5S41	CACAAAGCAGCATGTGACAAGAAAC	AA	TCC	TTTGCAACA	GCTCGACGT	AA	CATTGACAAGGCAGTTGCTCATGGG
Penk-1S4	AGTGATGCCTGGGACTATTCTATCT	AA	TCC	TTTGCA	GCTCGACGT	AA	CTGGAGGTATCCTATCTTCCCACGG
Penk-2S4	ATATAGCTCGTCCATCTTCTTCATG	AA	TCC	TTTGCA	GCTCGACGT	AA	GCCTCCGTACCGTTTCATGAAGCCT
Penk-3S4	AGTTGGCCAAGGTGTCTCCCTCATC	AA	TCC	TTTGCA	GCTCGACGT	AA	CATCCTTCTTCATGAAGCCGCCATA
Penk-4S4	CATACCTCTTGCTCATGTCTTCGTC	AA	TCC	TTTGCA	GCTCGACGT	AA	TGTTGGTGCTCTCTTGTTGGTGGCT
Penk-5S4	CACAAAGCAGCATGTGACAAGAAAC	AA	TCC	TTTGCA	GCTCGACGT	AA	CATTGACAAGGCAGTTGCTCATGGG
Penk-1S10	AGTGATGCCTGGGACTATTCTATCT	AA	AGC	CCATTAGAT	CGTCGGATG	AA	CTGGAGGTATCCTATCTTCCCACGG
Penk-2S10	ATATAGCTCGTCCATCTTCTTCATG	AA	AGC	CCATTAGAT	CGTCGGATG	AA	GCCTCCGTACCGTTTCATGAAGCCT
Penk-3S10	AGTTGGCCAAGGTGTCTCCCTCATC	AA	AGC	CCATTAGAT	CGTCGGATG	AA	CATCCTTCTTCATGAAGCCGCCATA
Penk-4S10	CATACCTCTTGCTCATGTCTTCGTC	AA	AGC	CCATTAGAT	CGTCGGATG	AA	TGTTGGTGCTCTCTTGTTGGTGGCT
Penk-5S10	CACAAAGCAGCATGTGACAAGAAAC	AA	AGC	CCATTAGAT	CGTCGGATG	AA	CATTGACAAGGCAGTTGCTCATGGG
Oxtr-1S23	AAGTGCTGTTCTAAAGAAGACAGGC	AA	TCG	AAGTCGTAT	GGGTGGTCG	AA	CAATGGTGGAGCATCCCTCGGGCCT
Oxtr-2S23	GGTATTTCAAAGGGACTCCAGAGTG	AA	TCG	AAGTCGTAT	GGGTGGTCG	AA	CAGTGACCAGACACAGGCAGAAAGC
Oxtr-3S23	CGCATACAGGTACATGTATGGGTTT	AA	TCG	AAGTCGTAT	GGGTGGTCG	AA	GCAAATACTGCAGCATAGCAGAAGA
Oxtr-4S23	GCTGGTGTCAACCAGCTTGCCCTAA	AA	TCG	AAGTCGTAT	GGGTGGTCG	AA	TGTGGATGTTGAACATGGGCACTGT
Oxtr-5S23	GCAGAATCGTGCTAGCTTCTCAAAG	AA	TCG	AAGTCGTAT	GGGTGGTCG	AA	CTGGGACAAGCTGGTGTGACACTGT
Oxtr-6S23	AGCAGGGAATCAGAGCACCTGCAAG	AA	TCG	AAGTCGTAT	GGGTGGTCG	AA	ACAGATGCTAACAGGCTACTGCCAT
Oxtr-7S23	ACCAGCTAGAGCTTTCATGGAGACT	AA	TCG	AAGTCGTAT	GGGTGGTCG	AA	CAACAGCAGGTTTCTATGCCCTCTG
Oxtr-8S23	GTGTGGAATCTGAGGAAGAAGAACA	AA	TCG	AAGTCGTAT	GGGTGGTCG	AA	GGTCACAGGACTGGGAGTGGGTGTT
Oxtr-9S23	GCACACTGACTCTTCATGGTCAGAA	AA	TCG	AAGTCGTAT	GGGTGGTCG	AA	ACGAAACATTCCAGAACATTCAGCT
Oxtr-10S23	TGCACTGACATCCTCCTGTGCTCTC	AA	TCG	AAGTCGTAT	GGGTGGTCG	AA	AAGTGATGGCTCAGATTTGTCCCAA
VG2-1S41	AATAGCTGCATGCAGCCCACGGGTT	AA	TCC	TTTGCAACA	GCTCGACGT	AA	CTAATAGGAGAATTGGTACACACAG
VG2-2S41	TCCAGCCTTACCAGATTTAAATTGT	AA	TCC	TTTGCAACA	GCTCGACGT	AA	TGGCTATGAAAGACGGATTCTGCGC
VG2-3S41	CTCTCGGTTGTCCTGCTTCTTCTCC	AA	TCC	TTTGCAACA	GCTCGACGT	AA	CACCCTGTAGATCTGTCCGAGGGAT
VG2-4S41	GCCTCCATTCTCCTGTGAGGTAGCA	AA	TCC	TTTGCAACA	GCTCGACGT	AA	GTAAGATTTGGTGGTACCGTAATTT
VG2-5S41	CGTGACAACTGCCACAGATTGCACT	AA	TCC	TTTGCAACA	GCTCGACGT	AA	ATGGGAATCTCATGGTCTGTTTTGA
